# *N*-(2-Bromo­benz­yl)-*N*′-(2-pyrid­yl)benzene-1,2-diamine

**DOI:** 10.1107/S1600536809039130

**Published:** 2009-10-03

**Authors:** Sudesh T. Manjare, Harkesh B. Singh, Ray J. Butcher

**Affiliations:** a Department of Chemistry, Indian Institute of Technology Bombay, Powai, Mumbai 400 076, India; b Department of Chemistry, Howard University, 525 College Street NW, Washington, DC 20059, USA

## Abstract

In the title compound, C_18_H_16_BrN_3_, mol­ecules are linked into dimers by co-operative inter­molecular N—H⋯N hydrogen bonding. Only one N—H group is involved in hydrogen bonding. The planes of the pyridine and bromo­phenyl rings are twisted by 61.49 (3) and 79.11 (8)°, respectively, from the plane of the central phenyl ring.

## Related literature

The title compound was isolated as part of a project to further investigate the chemistry of chalcogen–carbene compounds (Dutton *et al.*, 2007 ▶). The stability of imidazole-based carbenes depends very much on the nature of the substituents attached to the imidazole nitrogen atoms, see: Huynh *et al.* (2006 ▶); Kuhn *et al.* (1993 ▶). For bond lengths in analogous compounds, see: Albéniz *et al.* (2002 ▶); Denk *et al.* (2001 ▶). For details of the synthesis, see: Hahn *et al.* (2007 ▶).
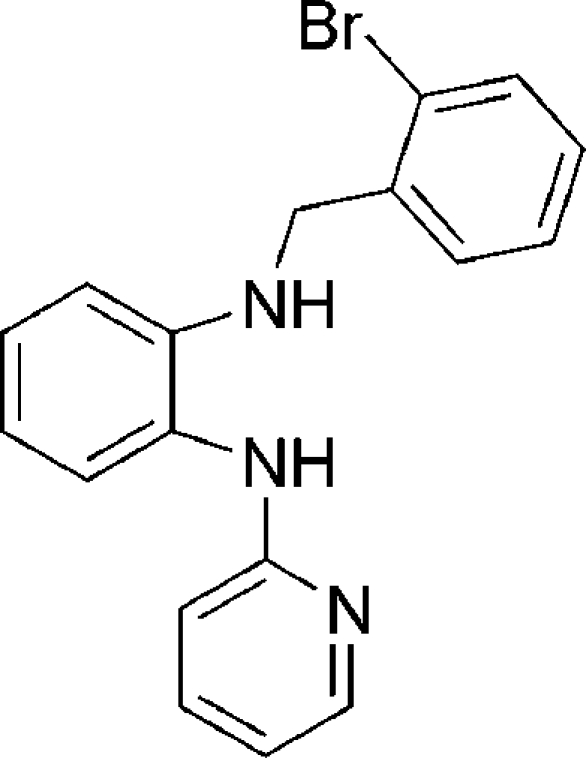


## Experimental

### 

#### Crystal data

C_18_H_16_BrN_3_
*M* *_r_* = 354.25
Triclinic, 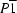

*a* = 7.9429 (5) Å
*b* = 9.5314 (8) Å
*c* = 11.0606 (8) Å
α = 98.741 (6)°
β = 90.727 (6)°
γ = 103.581 (6)°
*V* = 803.48 (10) Å^3^
*Z* = 2
Mo *K*α radiation
μ = 2.56 mm^−1^
*T* = 200 K
0.51 × 0.43 × 0.16 mm

#### Data collection

Oxford Diffraction Gemini R diffractometer
Absorption correction: multi-scan (*CrysAlis Pro*; Oxford Diffraction, 2009 ▶) *T*_min_ = 0.553, *T*_max_ = 1.000
8461 measured reflections
3249 independent reflections
2038 reflections with *I* > 2σ(*I*)
*R*_int_ = 0.042

#### Refinement

*R*[*F* ^2^ > 2σ(*F* ^2^)] = 0.039
*wR*(*F* ^2^) = 0.092
*S* = 0.89
3249 reflections
199 parameters
H-atom parameters constrained
Δρ_max_ = 0.59 e Å^−3^
Δρ_min_ = −0.48 e Å^−3^

### 

Data collection: *CrysAlis Pro* (Oxford Diffraction, 2009 ▶); cell refinement: *CrysAlis Pro*; data reduction: *CrysAlis Pro*; program(s) used to solve structure: *SHELXS97* (Sheldrick, 2008 ▶); program(s) used to refine structure: *SHELXL97* (Sheldrick, 2008 ▶); molecular graphics: *SHELXTL* (Sheldrick, 2008 ▶); software used to prepare material for publication: *SHELXTL*.

## Supplementary Material

Crystal structure: contains datablocks I, global. DOI: 10.1107/S1600536809039130/bt5071sup1.cif

Structure factors: contains datablocks I. DOI: 10.1107/S1600536809039130/bt5071Isup2.hkl

Additional supplementary materials:  crystallographic information; 3D view; checkCIF report

## Figures and Tables

**Table 1:** Hydrogen-bond geometry (Å, °)

*D*—H⋯*A*	*D*—H	H⋯*A*	*D*⋯*A*	*D*—H⋯*A*
N2—H2*A*⋯N3^i^	0.88	2.08	2.952 (3)	175

Symmetry code: (i) 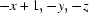.

**Fig. 1.**
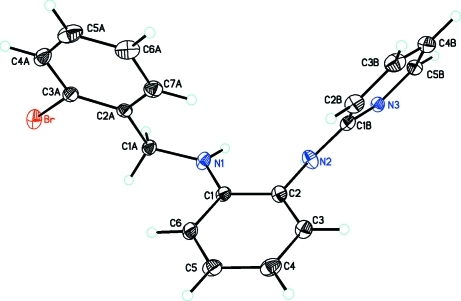
The molecular structure of C18H16BrN3 the showing the atom numbering scheme and 50% probability displacement ellipsoids.

**Fig. 2.**
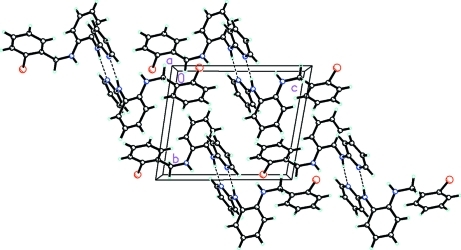
The molecular packing for C18H16BrN3 viewed down the a axis. The hydrogen bonding between N—H···N is shown by dashed lines.

**Fig. 3.**
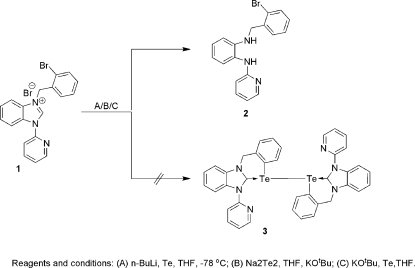
The formation of the title compound.

## supplementary crystallographic information

### Comment

The structure of the title compound, C_18_H_16_BrN_3_, (2), is shown below. Dimensions are available in the archived CIF.

Carbene compounds sometimes show unpredictable reactivity patterns and are subject to hydrolysis (Denk *et al.* 2001; Albéniz *et al.*, 2002). The stability of imidazole based carbenes depends very much on the nature of the substituents attached to the imidazole nitrogen atoms (Hahn *et al.*, 2007; Huynh *et al.* 2006).

The title compound was isolated as part of a project to further investigate the chemistry of chalcogen-carbene compounds (Dutton *et al.*, 2007), in particular tellurium-carbene chemistry with pyridine as a substituent on the nitrogen of the benzimidazole ring. However, in contrast with electron donating substituents such as *n*-butyl, and i-propyl, which lead to tellurium carbene formation, electron withdrawing groups such as phenyl and pyridyl result in hydrolysed products, such as the title compound. A repeated attempt to synthesize the pyridine substituted tellurone compound gave the title compound whose structure is reported here.

In (2) the bonds are in the usual ranges found for analogous compounds (Albéniz *et al.* 2002; Denk *et al.* 2001)).

The molecules are linked into dimers by cooperative intermolecular N—H···N hydrogen bonding. The two N—H moieties adopt different conformations with respect to the phenyl ring to which they are both attached. N1—H is only twisted by 18.0 (2)° from this plane. As a result of this coplanarity the hydrogen attached to N1 does not form any hydrogen bonds. N2—H, however, is twisted by 51.8 (2)° from this plane so as to participate in the intermolecular hydrogen bonding mentioned above. The planes of the pyridine and bromo-phenyl rings are twisted by 61.49 (3)° and 79.11 (8)° from the plane of the central phenyl ring.

The cleavage of carbene carbon from benzimidazole ring in the title compound may be due to: 1) destabilization of C=Te by the electron withdrawing group present on the benzimidazolium nitrogen, 2) crowding near to the carbene carbon. The exact mechanism is under investigation. This structural study has confirmed the cleavage of the carbene carbon.

### Experimental

In all cases, the starting benzylimidazoylium salt, 1, shown in scheme (1) was prepared using standard methods (Hahn *et al.* 2007). With the appropriate salt, the title compound could be made by three different methods: (a). In a round bottom flask the benzylimidazoylium salt 1 (1.0 mmol) was taken in THF (40 mL) under nitrogen atmosphere and of *n*-BuLi (2.0 mmol) was added at -78 °C, reaction mixture was stirred for 1-2 h. Then Te powder was added to the reaction mixture at room temperature, and stirred for 8-10 h. After completion of reaction, water (30 mL) was added and extracted with dichloromethane, dried over Na_2_SO_4_ and evaporated. The residue obtained was dissolved in toluene and small amount of petroleum ether was added to separate the residue from the solution. The solution was filtered, evaporated and the residue was dissolved in diethyl ether and a small amount of petroleum ether (60-80 °C) to afford the pure colorless product in 45% yield.

(b) The benzylimidazoylium salt 1 (1.0 mmol) was added to a brown solution of Na_2_Te_2_ (2.0 mmol) at room temperature under nitrogen atmosphere and the reaction mixture was stirred for 6-10 h at room temperature. Then KO*^t^*Bu (1.0 mmol) was added to the reaction mixture and stirred further for 5-7 h. After completion of reaction, the reaction was quenched by adding water (50 mL), and extracted with dichloromethane, dried over Na_2_SO_4_, and evaporated. The residue obtained was dissolved in toluene and small amount of petroleum ether was added to separate the residue from the solution. The solution was filtered and evaporated; the residue was dissolved in diethyl ether and a small amount of petroleum ether (60-80 °C) to afford the pure crystalline product.

(c) In a round bottom flask the benzylimidazoylium salt 1 (1.0 mmol) was taken in THF (40 mL) under nitrogen atmosphere and Te metal powder (1.0 mmol) was added, then KO*^t^*Bu (2.0 mmol) was added to the reaction mixture at -20 °C. The reaction mixture was stirred for 5-6 h. Then the reaction was quenched by adding water (50 mL), and extracted with dichloromethane, dried over Na_2_SO_4_, and evaporated. The residue obtained was dissolved in toluene and some petroleum ether was added to separate the residue from the solution. The solution was filtered and evaporated; the residue was dissolved in diethyl ether and small amount of petroleum ether (60-80 °C) to afford the pure product.

Mp 156-158 °C. ^1^H NMR (400 MHz, CDCl_3_): δ (ppm) 8.15 (m, ^1^H), 7.54 (dd, J = 7.6 Hz, J = 1.2 Hz, ^1^H), 7.43 (m, ^1^H), 7.32 (m, ^1^H), 7.23 (m, 2H), 7.11 (m, 2H), 6.71 (m, 2H), 6.61 (dd, J = 8 Hz, J = 1.2 Hz, ^1^H), 6.40 (m, ^1^H), 6.15 (s, ^1^H), 4.83 (d, J = 5.6 Hz, ^1^H), 4.41 (d, J = 6 Hz, 2H). ^13^C NMR (100 MHz, CDCl_3_): δ (ppm) 158.4, 148.4, 144.5, 138.1, 132.9, 128.9, 128.8, 127.8, 127.6, 127.4, 125.7, 123.4, 117.7, 114.6, 111.7, 107.4, 48.2. MS: m/z 353 [M]^+^, 355 [M+2]^+^ . Anal. Calcd. for C_18_H_16_BrN_3_ (%): C, 61.03; H, 4.55; N, 11.86. Found: C, 60.85; H, 4.55; N, 11.40.

### Refinement

H atoms were placed in geometrically idealized positions and constrained to ride on their parent atoms with C—H distances of 0.95 and 0.99 Å *U*_iso_(H) = 1.2*U*_eq_(C). The H attached to N was idealized with a distance of 0.88 Å.

### Crystal data

**Table:**

C_18_H_16_BrN_3_	*Z* = 2
*M**_r_* = 354.25	*F*(000) = 360
Triclinic, *P*1	*D*_x_ = 1.464 Mg m^−^^3^
Hall symbol: -P 1	Mo *K*α radiation, λ = 0.71073 Å
*a* = 7.9429 (5) Å	Cell parameters from 3047 reflections
*b* = 9.5314 (8) Å	θ = 4.7–34.8°
*c* = 11.0606 (8) Å	µ = 2.56 mm^−^^1^
α = 98.741 (6)°	*T* = 200 K
β = 90.727 (6)°	Irregular plate, colorless
γ = 103.581 (6)°	0.51 × 0.43 × 0.16 mm
*V* = 803.48 (10) Å^3^	

### Data collection

**Table:**

Oxford Diffraction Gemini R diffractometer	3249 independent reflections
Radiation source: Enhance (Mo) X-ray Source	2038 reflections with *I* > 2σ(*I*)
graphite	*R*_int_ = 0.042
Detector resolution: 10.5081 pixels mm^-1^	θ_max_ = 26.4°, θ_min_ = 4.7°
ω scans	*h* = −9→9
Absorption correction: multi-scan (*CrysAlis PRO*; Oxford Diffraction, 2009)	*k* = −11→11
*T*_min_ = 0.553, *T*_max_ = 1.000	*l* = −13→13
8461 measured reflections	

### Refinement

**Table:**

Refinement on *F*^2^	Primary atom site location: structure-invariant direct methods
Least-squares matrix: full	Secondary atom site location: difference Fourier map
*R*[*F*^2^ > 2σ(*F*^2^)] = 0.039	Hydrogen site location: inferred from neighbouring sites
*wR*(*F*^2^) = 0.092	H-atom parameters constrained
*S* = 0.89	*w* = 1/[σ^2^(*F*_o_^2^) + (0.052*P*)^2^] where *P* = (*F*_o_^2^ + 2*F*_c_^2^)/3
3249 reflections	(Δ/σ)_max_ = 0.001
199 parameters	Δρ_max_ = 0.59 e Å^−^^3^
0 restraints	Δρ_min_ = −0.48 e Å^−^^3^

### Special details

**Table:**

Geometry. All e.s.d.'s (except the e.s.d. in the dihedral angle between two l.s. planes) are estimated using the full covariance matrix. The cell e.s.d.'s are taken into account individually in the estimation of e.s.d.'s in distances, angles and torsion angles; correlations between e.s.d.'s in cell parameters are only used when they are defined by crystal symmetry. An approximate (isotropic) treatment of cell e.s.d.'s is used for estimating e.s.d.'s involving l.s. planes.
Refinement. Refinement of *F*^2^ against ALL reflections. The weighted *R*-factor *wR* and goodness of fit *S* are based on *F*^2^, conventional *R*-factors *R* are based on *F*, with *F* set to zero for negative *F*^2^. The threshold expression of *F*^2^ > σ(*F*^2^) is used only for calculating *R*-factors(gt) *etc*. and is not relevant to the choice of reflections for refinement. *R*-factors based on *F*^2^ are statistically about twice as large as those based on *F*, and *R*- factors based on ALL data will be even larger.

### Fractional atomic coordinates and isotropic or equivalent isotropic displacement parameters (Å^2^)

**Table:**

	*x*	*y*	*z*	*U*_iso_*/*U*_eq_	
Br	0.83487 (5)	0.00876 (4)	0.69232 (3)	0.05856 (16)	
N1	0.7358 (3)	0.1251 (3)	0.31573 (19)	0.0433 (6)	
H1A	0.6776	0.0542	0.2595	0.052*	
N2	0.5888 (3)	0.1863 (3)	0.1091 (2)	0.0405 (6)	
H2A	0.6118	0.1008	0.0849	0.049*	
N3	0.3381 (3)	0.0980 (2)	−0.01114 (19)	0.0326 (5)	
C1	0.7931 (4)	0.2636 (3)	0.2846 (2)	0.0351 (7)	
C2	0.7198 (4)	0.2970 (3)	0.1804 (2)	0.0348 (7)	
C3	0.7745 (4)	0.4348 (3)	0.1484 (3)	0.0408 (7)	
H3A	0.7240	0.4563	0.0772	0.049*	
C4	0.9013 (4)	0.5417 (4)	0.2185 (3)	0.0477 (8)	
H4A	0.9353	0.6370	0.1975	0.057*	
C5	0.9773 (4)	0.5079 (4)	0.3189 (3)	0.0481 (9)	
H5A	1.0669	0.5799	0.3661	0.058*	
C6	0.9258 (4)	0.3714 (3)	0.3522 (2)	0.0467 (8)	
H6A	0.9807	0.3501	0.4217	0.056*	
C1A	0.7671 (4)	0.0915 (3)	0.4359 (2)	0.0386 (7)	
H1AA	0.8930	0.1249	0.4575	0.046*	
H1AB	0.7348	−0.0160	0.4317	0.046*	
C2A	0.6705 (3)	0.1589 (3)	0.5388 (2)	0.0342 (7)	
C3A	0.6902 (4)	0.1324 (3)	0.6583 (2)	0.0384 (7)	
C4A	0.6043 (4)	0.1895 (4)	0.7542 (3)	0.0487 (9)	
H4AA	0.6192	0.1680	0.8342	0.058*	
C5A	0.4973 (5)	0.2775 (4)	0.7325 (3)	0.0563 (10)	
H5AA	0.4387	0.3185	0.7981	0.068*	
C6A	0.4744 (4)	0.3068 (4)	0.6165 (3)	0.0546 (9)	
H6AA	0.3992	0.3672	0.6018	0.066*	
C7A	0.5616 (4)	0.2476 (3)	0.5202 (3)	0.0441 (8)	
H7AA	0.5456	0.2690	0.4403	0.053*	
C1B	0.4294 (4)	0.2041 (3)	0.0757 (2)	0.0343 (7)	
C2B	0.3615 (4)	0.3199 (3)	0.1284 (3)	0.0434 (8)	
H2BA	0.4278	0.3943	0.1892	0.052*	
C3B	0.1994 (4)	0.3253 (4)	0.0920 (3)	0.0492 (8)	
H3BA	0.1509	0.4029	0.1282	0.059*	
C4B	0.1052 (4)	0.2177 (4)	0.0020 (3)	0.0495 (8)	
H4BA	−0.0076	0.2202	−0.0258	0.059*	
C5B	0.1800 (4)	0.1081 (4)	−0.0453 (3)	0.0416 (7)	
H5BA	0.1155	0.0336	−0.1068	0.050*	

### Atomic displacement parameters (Å^2^)

**Table:**

	*U*^11^	*U*^22^	*U*^33^	*U*^12^	*U*^13^	*U*^23^
Br	0.0621 (3)	0.0737 (3)	0.0418 (2)	0.01693 (18)	−0.00365 (15)	0.01454 (16)
N1	0.0608 (17)	0.0325 (16)	0.0236 (12)	−0.0093 (12)	−0.0039 (11)	−0.0031 (10)
N2	0.0443 (15)	0.0301 (15)	0.0423 (13)	0.0096 (12)	−0.0114 (12)	−0.0092 (11)
N3	0.0360 (14)	0.0304 (14)	0.0282 (12)	0.0038 (10)	−0.0026 (10)	0.0016 (10)
C1	0.0394 (17)	0.0335 (18)	0.0242 (14)	−0.0033 (13)	0.0026 (12)	−0.0020 (12)
C2	0.0414 (17)	0.0313 (18)	0.0255 (14)	0.0038 (13)	−0.0022 (12)	−0.0071 (12)
C3	0.0469 (18)	0.037 (2)	0.0376 (16)	0.0091 (15)	0.0018 (14)	0.0042 (14)
C4	0.056 (2)	0.036 (2)	0.0449 (18)	0.0000 (15)	0.0111 (16)	0.0032 (14)
C5	0.052 (2)	0.042 (2)	0.0337 (16)	−0.0149 (15)	0.0044 (15)	−0.0058 (14)
C6	0.052 (2)	0.050 (2)	0.0245 (14)	−0.0113 (16)	−0.0044 (13)	−0.0005 (14)
C1A	0.0429 (18)	0.0388 (19)	0.0280 (14)	−0.0001 (13)	0.0022 (12)	0.0020 (12)
C2A	0.0305 (16)	0.0300 (17)	0.0330 (15)	−0.0077 (13)	0.0005 (12)	0.0001 (12)
C3A	0.0389 (17)	0.0340 (18)	0.0325 (15)	−0.0070 (13)	0.0020 (13)	0.0000 (12)
C4A	0.055 (2)	0.046 (2)	0.0322 (16)	−0.0092 (17)	0.0068 (15)	−0.0018 (14)
C5A	0.060 (2)	0.040 (2)	0.060 (2)	0.0016 (18)	0.0256 (18)	−0.0052 (17)
C6A	0.051 (2)	0.041 (2)	0.072 (2)	0.0117 (16)	0.0156 (18)	0.0075 (17)
C7A	0.0418 (18)	0.037 (2)	0.0497 (18)	0.0007 (15)	0.0040 (14)	0.0094 (14)
C1B	0.0397 (17)	0.0336 (18)	0.0284 (14)	0.0055 (13)	0.0060 (13)	0.0058 (12)
C2B	0.052 (2)	0.0335 (19)	0.0420 (17)	0.0080 (15)	0.0057 (15)	−0.0006 (13)
C3B	0.054 (2)	0.042 (2)	0.058 (2)	0.0196 (17)	0.0179 (17)	0.0119 (16)
C4B	0.0390 (18)	0.060 (2)	0.054 (2)	0.0136 (17)	0.0071 (16)	0.0197 (17)
C5B	0.0356 (18)	0.047 (2)	0.0394 (16)	0.0036 (15)	−0.0004 (14)	0.0095 (14)

### Geometric parameters (Å, °)

**Table:**

Br—C3A	1.900 (3)	C1A—H1AA	0.9900
N1—C1	1.389 (4)	C1A—H1AB	0.9900
N1—C1A	1.446 (3)	C2A—C7A	1.377 (4)
N1—H1A	0.8800	C2A—C3A	1.396 (4)
N2—C1B	1.370 (3)	C3A—C4A	1.380 (4)
N2—C2	1.423 (3)	C4A—C5A	1.369 (5)
N2—H2A	0.8800	C4A—H4AA	0.9500
N3—C5B	1.336 (4)	C5A—C6A	1.372 (5)
N3—C1B	1.347 (3)	C5A—H5AA	0.9500
C1—C2	1.397 (4)	C6A—C7A	1.398 (4)
C1—C6	1.401 (4)	C6A—H6AA	0.9500
C2—C3	1.384 (4)	C7A—H7AA	0.9500
C3—C4	1.383 (4)	C1B—C2B	1.393 (4)
C3—H3A	0.9500	C2B—C3B	1.359 (4)
C4—C5	1.372 (4)	C2B—H2BA	0.9500
C4—H4A	0.9500	C3B—C4B	1.384 (5)
C5—C6	1.377 (4)	C3B—H3BA	0.9500
C5—H5A	0.9500	C4B—C5B	1.361 (4)
C6—H6A	0.9500	C4B—H4BA	0.9500
C1A—C2A	1.526 (4)	C5B—H5BA	0.9500
			
C1—N1—C1A	123.2 (2)	C7A—C2A—C1A	122.8 (3)
C1—N1—H1A	118.4	C3A—C2A—C1A	120.4 (3)
C1A—N1—H1A	118.4	C4A—C3A—C2A	122.6 (3)
C1B—N2—C2	124.4 (2)	C4A—C3A—Br	117.6 (2)
C1B—N2—H2A	117.8	C2A—C3A—Br	119.8 (2)
C2—N2—H2A	117.8	C5A—C4A—C3A	119.1 (3)
C5B—N3—C1B	117.5 (2)	C5A—C4A—H4AA	120.4
N1—C1—C2	119.3 (2)	C3A—C4A—H4AA	120.4
N1—C1—C6	122.5 (3)	C4A—C5A—C6A	120.3 (3)
C2—C1—C6	118.1 (3)	C4A—C5A—H5AA	119.9
C3—C2—C1	120.1 (2)	C6A—C5A—H5AA	119.9
C3—C2—N2	121.5 (3)	C5A—C6A—C7A	120.0 (3)
C1—C2—N2	118.4 (3)	C5A—C6A—H6AA	120.0
C4—C3—C2	121.1 (3)	C7A—C6A—H6AA	120.0
C4—C3—H3A	119.5	C2A—C7A—C6A	121.3 (3)
C2—C3—H3A	119.5	C2A—C7A—H7AA	119.4
C5—C4—C3	118.9 (3)	C6A—C7A—H7AA	119.4
C5—C4—H4A	120.5	N3—C1B—N2	115.0 (2)
C3—C4—H4A	120.5	N3—C1B—C2B	121.4 (3)
C4—C5—C6	121.1 (3)	N2—C1B—C2B	123.7 (3)
C4—C5—H5A	119.4	C3B—C2B—C1B	119.3 (3)
C6—C5—H5A	119.4	C3B—C2B—H2BA	120.4
C5—C6—C1	120.6 (3)	C1B—C2B—H2BA	120.4
C5—C6—H6A	119.7	C2B—C3B—C4B	119.8 (3)
C1—C6—H6A	119.7	C2B—C3B—H3BA	120.1
N1—C1A—C2A	115.7 (3)	C4B—C3B—H3BA	120.1
N1—C1A—H1AA	108.4	C5B—C4B—C3B	117.5 (3)
C2A—C1A—H1AA	108.4	C5B—C4B—H4BA	121.2
N1—C1A—H1AB	108.4	C3B—C4B—H4BA	121.2
C2A—C1A—H1AB	108.4	N3—C5B—C4B	124.5 (3)
H1AA—C1A—H1AB	107.4	N3—C5B—H5BA	117.8
C7A—C2A—C3A	116.8 (2)	C4B—C5B—H5BA	117.8
			
C1A—N1—C1—C2	−162.8 (3)	C7A—C2A—C3A—Br	179.0 (2)
C1A—N1—C1—C6	18.9 (4)	C1A—C2A—C3A—Br	−1.2 (3)
N1—C1—C2—C3	179.5 (2)	C2A—C3A—C4A—C5A	−1.0 (5)
C6—C1—C2—C3	−2.0 (4)	Br—C3A—C4A—C5A	−179.2 (2)
N1—C1—C2—N2	−0.4 (4)	C3A—C4A—C5A—C6A	0.8 (5)
C6—C1—C2—N2	178.0 (2)	C4A—C5A—C6A—C7A	−0.6 (5)
C1B—N2—C2—C3	−52.3 (4)	C3A—C2A—C7A—C6A	−0.6 (4)
C1B—N2—C2—C1	127.6 (3)	C1A—C2A—C7A—C6A	179.6 (3)
C1—C2—C3—C4	−0.2 (4)	C5A—C6A—C7A—C2A	0.4 (5)
N2—C2—C3—C4	179.7 (3)	C5B—N3—C1B—N2	178.6 (2)
C2—C3—C4—C5	2.1 (4)	C5B—N3—C1B—C2B	0.2 (4)
C3—C4—C5—C6	−1.8 (5)	C2—N2—C1B—N3	167.2 (2)
C4—C5—C6—C1	−0.4 (5)	C2—N2—C1B—C2B	−14.5 (4)
N1—C1—C6—C5	−179.3 (3)	N3—C1B—C2B—C3B	0.5 (4)
C2—C1—C6—C5	2.3 (4)	N2—C1B—C2B—C3B	−177.8 (3)
C1—N1—C1A—C2A	68.9 (3)	C1B—C2B—C3B—C4B	−1.1 (5)
N1—C1A—C2A—C7A	−0.8 (4)	C2B—C3B—C4B—C5B	1.0 (5)
N1—C1A—C2A—C3A	179.4 (2)	C1B—N3—C5B—C4B	−0.3 (4)
C7A—C2A—C3A—C4A	0.9 (4)	C3B—C4B—C5B—N3	−0.3 (5)
C1A—C2A—C3A—C4A	−179.3 (3)		

### Hydrogen-bond geometry (Å, °)

**Table:**

*D*—H···*A*	*D*—H	H···*A*	*D*···*A*	*D*—H···*A*
N2—H2A···N3^i^	0.88	2.08	2.952 (3)	175

Symmetry codes: (i) −*x*+1, −*y*, −*z*.
